# A Case of Horizontal Gaze Palsy With Progressive Scoliosis and G6PD Deficiency in a Child

**DOI:** 10.7759/cureus.86551

**Published:** 2025-06-22

**Authors:** Parmeet Kaur, Sangita Yadav

**Affiliations:** 1 Pediatrics and Child Health, Hamdard Institute of Medical Sciences, New Delhi, IND; 2 Paediatrics and Child Health, Hamdard Institute of Medical Sciences, New Delhi, IND

**Keywords:** glucose-6-phosphate-dehydrogenase deficiency (g6pd), horizontal gaze palsy, pediatrics, pediatric scoliosis, robo 3 gene mutation

## Abstract

Horizontal gaze palsy with progressive scoliosis (HGPPS) is a rare autosomal recessive disease associated with mutation in the Roundabout-3 (ROBO3) gene (chromosome 11q23-25). Here, we present case report of a 7-year old male child born out of consanguineous marriage with history of similar condition in paternal uncle. The child had typical findings of HGPPS, i.e., horizontal gaze palsy, scoliosis, and characteristic findings on MRI associated with homozygous c.575G>A (p.Gly192Asp) mutation in ROBO3 gene. Additionally, mutation in G6PD gene was also observed in this patient, hypothesizing possible association between the two.

## Introduction

Horizontal gaze palsy with progressive scoliosis (HGPPS; Online Mendelian Inheritance in Man (OMIM) 607313) is a rare genetic disorder with autosomal recessive mode of inheritance [1]. It is characterized by the congenital static limitation of horizontal gaze with preservation of vertical gaze and progressive scoliosis that usually begins in childhood.

The disorder was first noted by Dretakis and Kondoyannis in five children of two families in year 1974 and its first neurological description was given by Crisfield [2,3]. This disorder is associated with mutations in ROBO3 genes located on chromosome 11q23-25, which encodes for protein that shares homology with a member of the Roundabout (ROBO) gene family [1]. This protein has a role in neurite outgrowth, growth cone guidance and regulation of hind brain axon midline crossing [4].

Function loss in this gene results in uncrossed pathways of corticospinal and dorsal columnn medial leminiscus. Horizontal gaze palsy can occur due to defects in the abducens nuclei containing ipsilateral and interneuronal motor neurons that project contralaterally, or supranuclear control regions, i.e., paramedian pontine reticular formation that controls abducens and oculomotor nuclei [4]. Magnetic resonance (MR) neuroimaging findings of HGPPS was first discussed by Pieh et al., where they had reported underdevelopment of both the pons and medulla, absence of the facial colliculi and butterfly-like configuration of the medulla oblongata [5]. However, they did not report split pons sign, i.e., midline division on ridge part of the pons. It was were reported in subsequent studies.

In this case report, we present the phenotypic ophthalmologic and neuroimaging characteristics of 7-year-old male patient with ROBO3 gene mutation along with G6PD deficiency. Written consent for this case report and patient's identity disclosure was obtained from the parents of the patient.

## Case presentation

A 7-year-old male patient, born of third-degree consanguineous marriage, attended the outpatient department (OPD) at HAHC Hospital with complaints of inability to move both eyes horizontally and deformity in spine. Parents declared that at five months, he had torticollis, which got corrected on its own. At the age of three, the mother noticed that the child has horizontal gaze limitation and also deviation of spine towards one side. On inquiry, it was found that there is a similar history in a paternal uncle, who also had progressive spine deformity since childhood progressing to difficulty in ambulation. Additionally, child had history of jaundice at six months of age for which he was admitted, but the cause had not been evaluated at that time.

At the time of presentation, neurological examination revealed normal mental status tone, and reflexes. Power and sensory evaluation of all the limbs were normal. Patient was not able to move his eyes sideways suggesting bilateral sixth cranial nerve palsy. Neurodevelopmental examination of the child was normal with developmental quotient (DQ) of 98%. All milestones were achieved at appropriate age except head control, which was attained at seven months. Psychological assessment showed social quotient (SQ) of 106 as per Vineland Social Maturity Scale.

**Figure 1:**
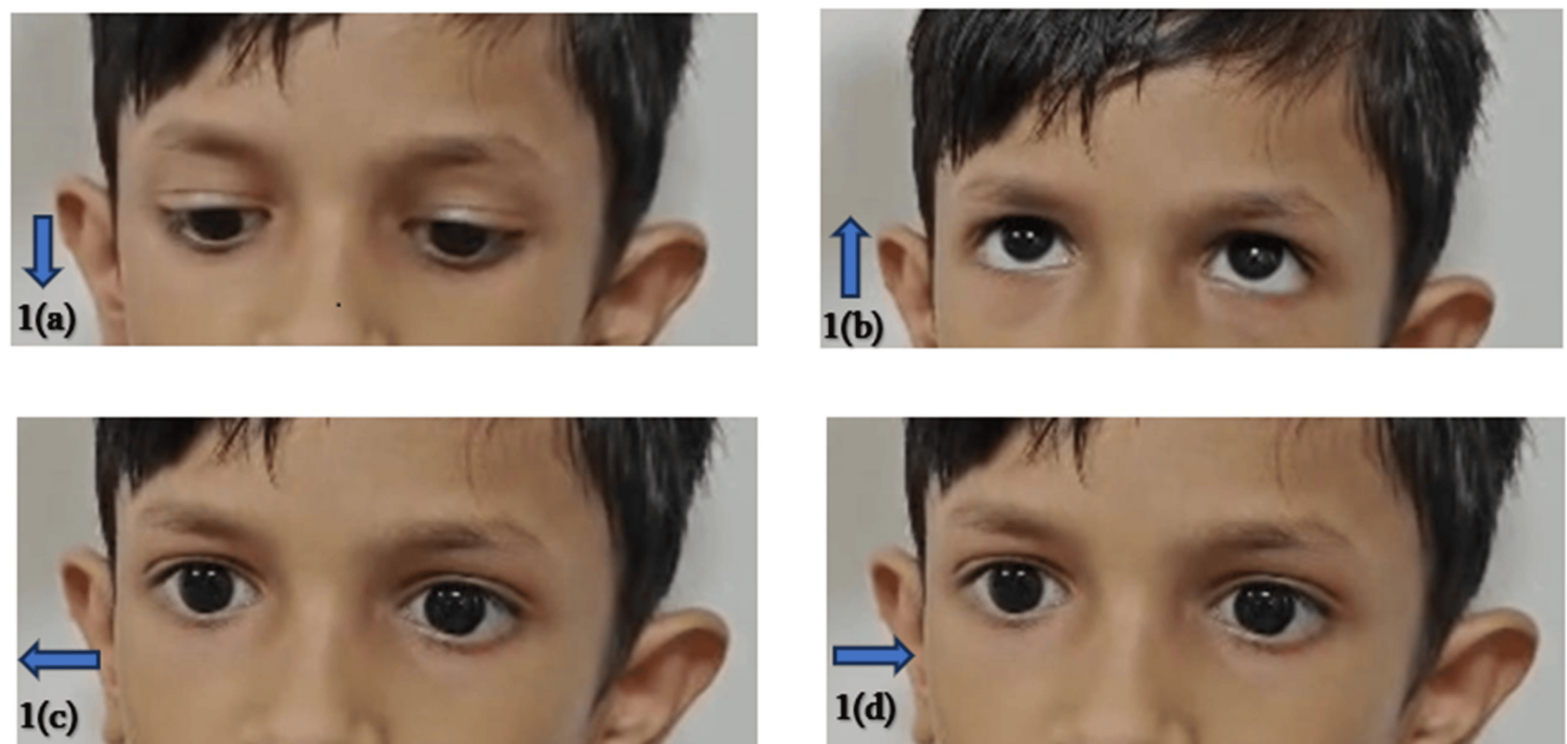
Cardinal directions of gaze (indicated by arrows) in this patient. (a) and (b): normal vertical gaze; (c) and (d): horizontal gaze palsy.

Ophthalmological examination showed normal vertical eye movements with absent horizontal movements (Figure 1).

He had no abnormal head position. Lens was clear, and pupillary reactions, corneal sensations, and Bell’s phenomenon were normal. Examination of anterior segment and posterior segment was normal as well. Post Mydriatic Test (PMT) showed -1.0×70 D 6/18 in right eye and -0.75x150D 6/18 in left eye. Non-contact tonometry was 11 mmHg and 10 mmHg in right and left eye, respectively. Forced duction test was negative and there was no diplopia.

**Figure 2:**
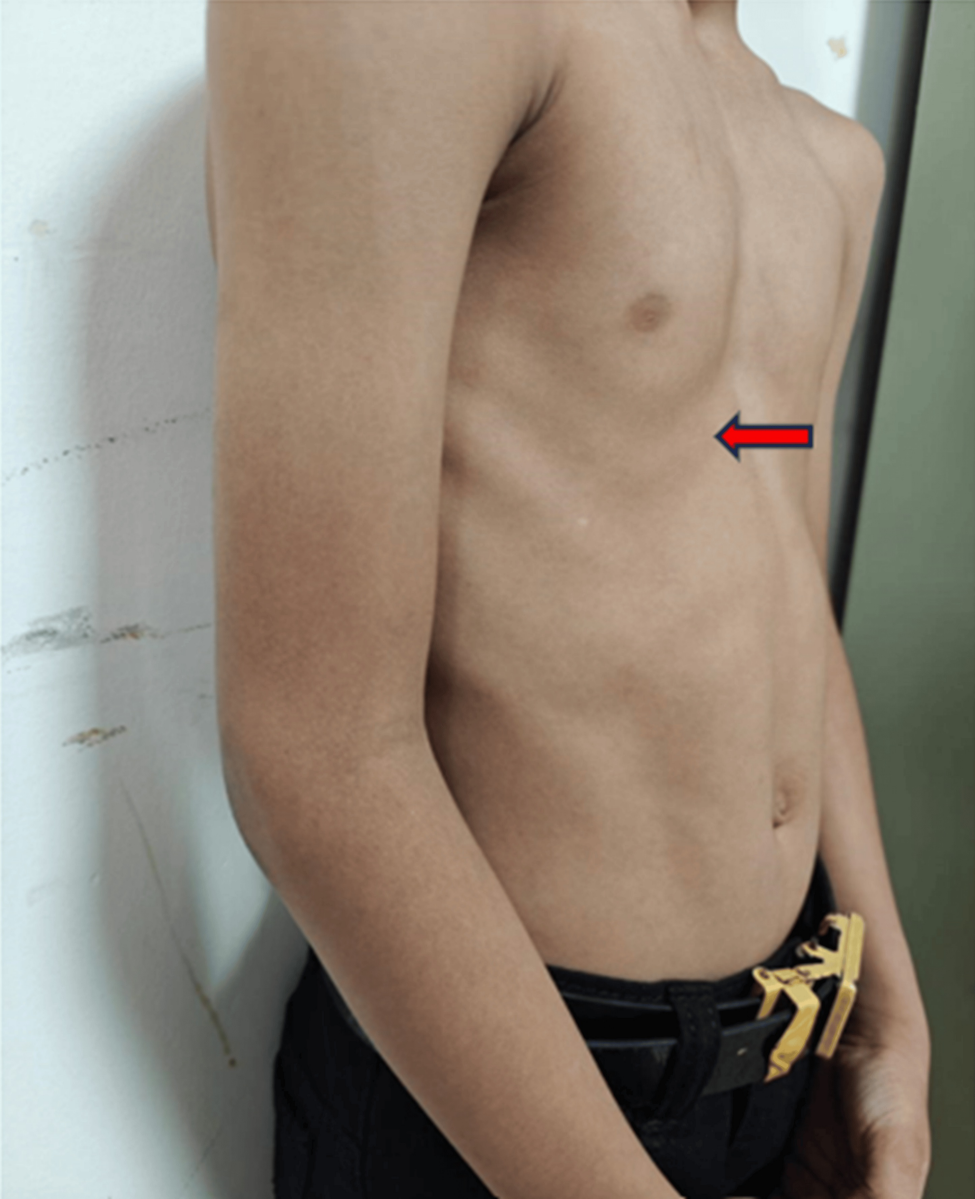
Pectus excavatum deformity (arrow)

**Figure 3:**
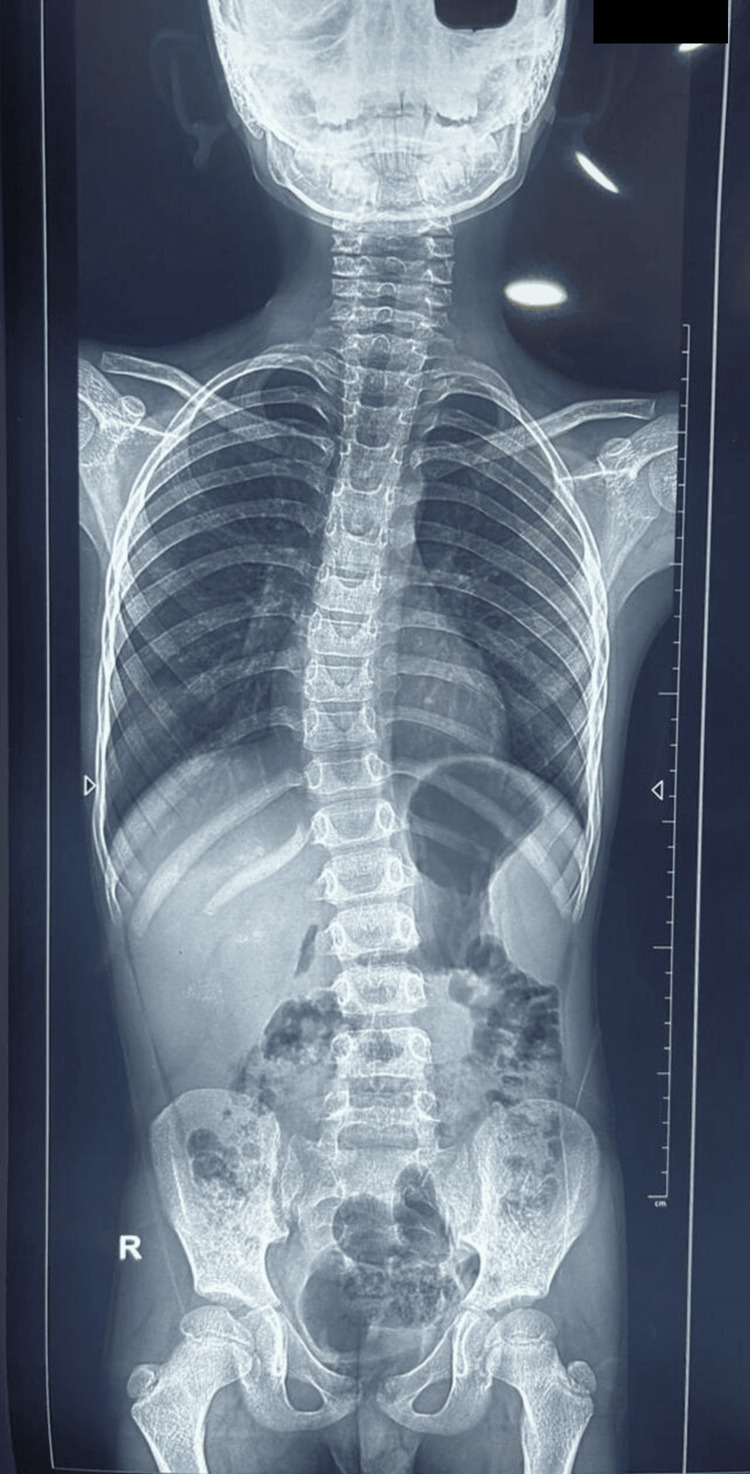
Scoliotic deformity is 8.9˚ in X-ray spine

Child had right-sided scoliosis along with pectus excavatum deformity as shown in Figure 2. Cobb’s angle of the scoliotic deformity was 8.9˚ as depicted in X-ray of spine (Figure 3).

**Figure 4:**
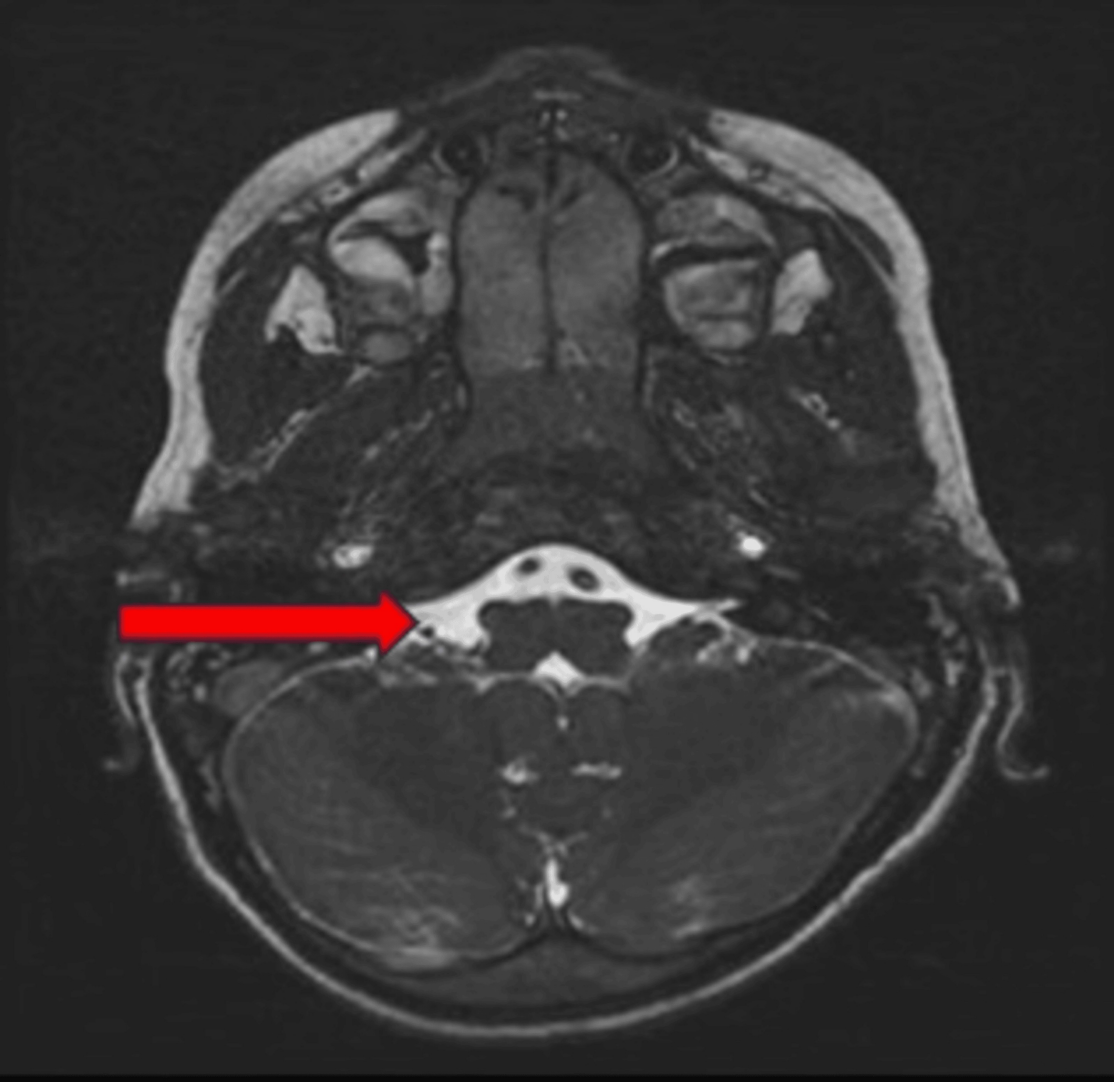
Axial T2-weighted MRI showing rectangular shaped medulla due to mild reduction in volume leading to “butterfly sign” (arrow)

**Figure 5:**
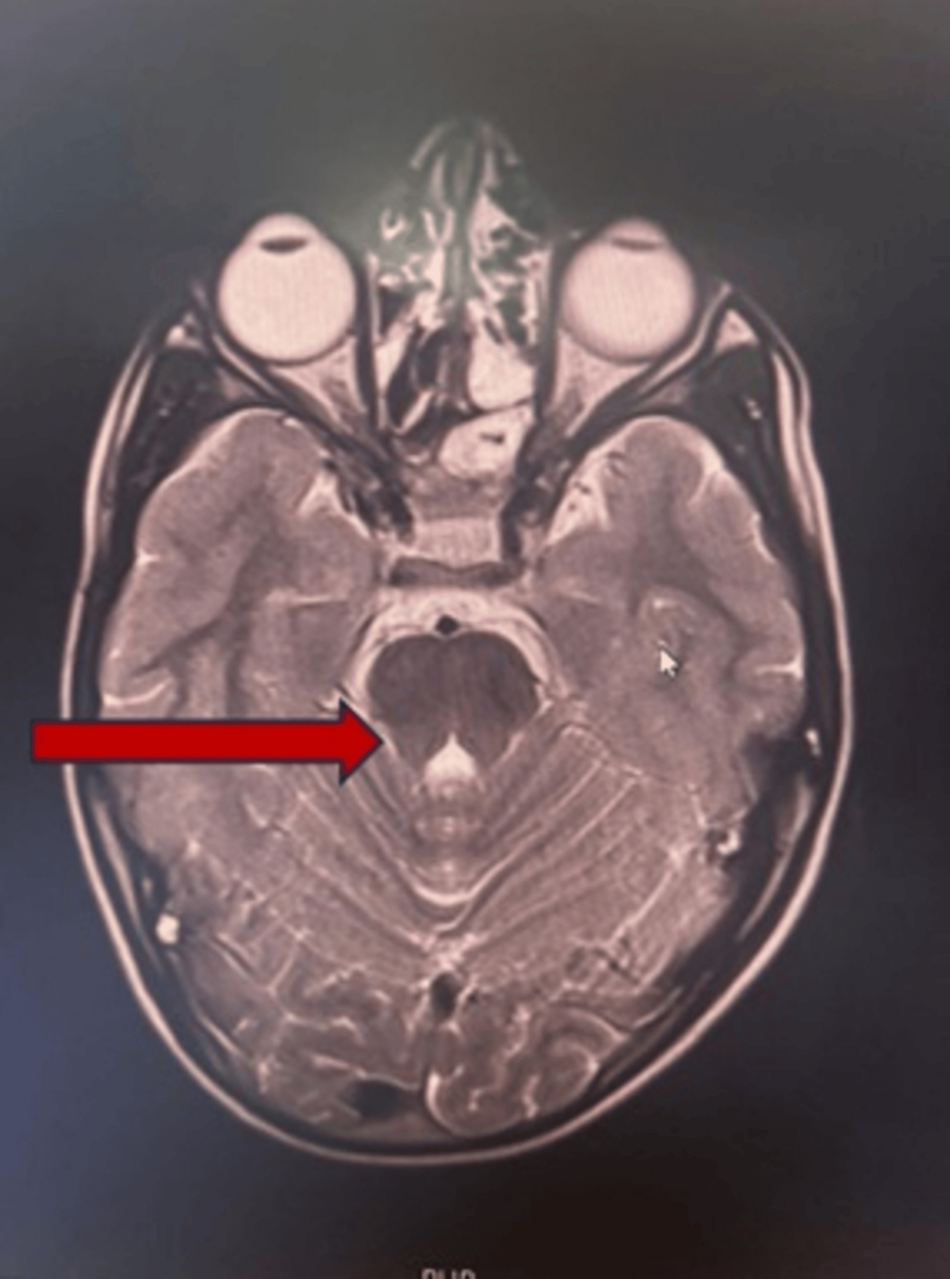
Axial T2-weighted MRI showing partial split in the dorsal aspect of pons (split pons sign; arrow)

MRI brain showed mild reduction in the volume of the medulla, which appears rectangular and butterfly shaped in appearance (Figure 4). It also showed abnormal development of the abducens nuclei with absence of facial colliculus. There is also mild reduction in the volume of the pons with partial split in the dorsal aspect of pons causing tenting of the fourth ventricle (Figure 5).

**Figure 6:**
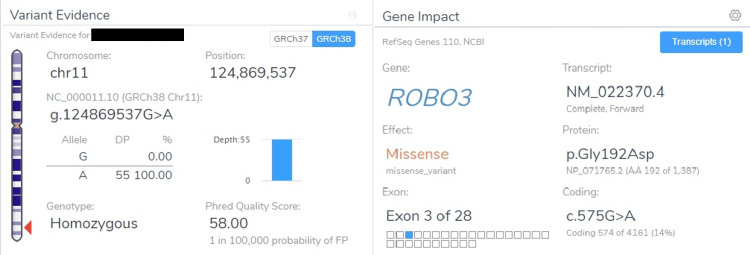
Whole genome sequencing report showing ROBO 3 gene mutation Based on above evidence, this variant (ROBO3:c.575G>A) is classified as uncertain significance variant.

Whole genome sequencing showed a homozygous variant in Exon 3 of gene ROBO3. A missense variant of “G” to “A” detected at nucleotide position 575 leading to change in amino acid sequences from glycine to aspartic acid at codon 192 (Figure 6).

Genomic studies also showed a hemizygous variant in Exon 3 in the G6PD gene on chromosome Xq28, which is likely pathogenic (Figure 7).

**Table 1:** Genetic test result showing G6PD deficiency VAF: Variant allele frequency; OMIM: Online Mendelian Inheritance in Man

Gene & Transcript	Location	Variant & Depth/VAF	Zygosity/Inheritance	OMIM Phenotype	Clinical Significance
G6PD (-)	Exon 3	c.131C>G	Hemizygous/X-linked	Anemia, nonspherocytic hemolytic, due to G6PD deficiency	Likely pathogenic (PM1, PP2, PS1, PM5)
NM_001042351.3		(p.Ala44Gly)			
(chrX:g.154536168G>C)		43x/100%			

The patient was advised to use Boston brace for correcting scoliosis and is on regular physiotherapy. For squint correction, the child is on regular follow up with ophthalmology department.

## Discussion

ROBO3 gene has an important role in cell migration and axonal midline crossing of hindbrain. A total of 25 case reports and case series have been published between 1998 and 2019 [6]. Most of the studies have shown that the disease is autosomal recessive in inheritance. Systematic review by Pinero-Pinto et al. found that 48.4% of HGPSS cases had consanguineous marriage [6]. In our study, the child and his paternal uncle were affected. Both affected family members have developed the symptoms in their childhood, which progressed over time.

In ophthalmological evaluation of our patient, the typical finding of horizontal gaze palsy with preserved vertical gaze was noted, a finding consistently reported in all previously documented cases. The scoliosis that was observed in these cases was progressive. However, we cannot comment on the progressive nature of scoliotic deformity in our patient as the disease was in an early stage and requires further follow-up. The patient has an additional skeletal deformity of the sternum (pectus excavatum), possibly for accommodating scoliotic defect, or it could be another manifestation of the disease. MRI of our patient showed typical findings as described in the literature. Figures 4 and 5 shows the butterfly sign and split pons sign, respectively, which were also reported in some studies [7-9].

The variant detected in our patient is ROBO3 : c.575G>A, which is classified as an uncertain significance variant, a novel mutation. Another striking finding in whole exon sequencing is a pathological mutation in the G6PD gene. This association has not been described in any literature till date.

## Conclusions

Early diagnosis of HGPPS is crucial to prevent the progression of scoliotic deformity. Timely surgical intervention can help improve the quality of life. This case report acknowledges the existing literature on HGPPS along with additional findings (G6PD gene mutation) that could be related to this condition.
